# Identification and Development of Inflammatory Response–Related Genes Signature Associated With Prognosis Evaluation and Immune Status of Bladder Cancer

**DOI:** 10.3389/fcell.2022.837849

**Published:** 2022-03-03

**Authors:** Haoxiang Zheng, Weihan Luo, Yuqing Li, Guoyu Peng, Dewang Zhou, Dongdong Tang, Jiwen Cheng, Song Wu

**Affiliations:** ^1^ Luohu Clinical Medicine School, Shantou University Medical College, Shantou University, Shantou, China; ^2^ Institute of Urology, The Third Affiliated Hospital of Shenzhen University (Luohu Hospital Group), Shenzhen University, Shenzhen, China; ^3^ Department of Urology, The First Affiliated Hospital of Guangxi Medical University, Nanning, China; ^4^ Kobilka Institute of Innovative Drug Discovery, School of Life and Health Sciences, Chinese University of Hong Kong, Shenzhen, China; ^5^ Department of Urology, Lanzhou University Second Hospital, Lanzhou, China; ^6^ Health Science Center, South China Hospital, Shenzhen University, Shenzhen, China

**Keywords:** bladder cancer, inflammatory response, prognosis, immune status, drug sensitivity, tumor microenvironment

## Abstract

**Background:** Bladder urothelial carcinoma (BLCA) is one of the most common malignant tumors with high morbidity and recurrence rate. The study aims to establish a prediction model to elaborate the relation between inflammatory response and prognosis of BLCA and thus to evaluate the potential prognostic value of inflammatory response–related genes (IRGs) in therapeutic choices.

**Methods:** The study utilized the gene expression profiles from the The Cancer Genome Atlas and Gene Expression Omnibus (GSE32894) datasets. Differentially expressed IRGs between normal and tumor tissues were identified, and 10 of them were correlated with overall survival (OS) (*p* < 0.05). Then, the LASSO–Cox regression analysis was applied to optimize the signature. RNA sequencing data of patients with BLCA from GSE32894 were applied as a validation set. Cox regression analyses of the seven-gene signature were performed to examine the efficiency of signature in predicting prognosis. Receiver operating characteristic curve analysis was applied to measure the predictive performance of the risk score for OS. Analysis of independent prognostic factors, downstream functional enrichment, drug sensitivity, and immune features were included in this study.

**Results:** The IRG signature (LDLR, ROS1, MMP14, TNFAIP6, MYC, PTGER4, and RIPK2) was used to divide patients into high- and low-risk groups. Cox regression analyses revealed that the risk score was an independent predictive factor. Functional enrichment analysis revealed that genes were enriched in prognosis-related molecular functions and immune-related biological processes. Drug sensitivity and tumor microenvironment correlation analysis indicated that the signature was related to immunotherapy effect.

**Conclusion:** The study defined a new prognostic signature consisting of seven IRGs, which could effectively predict the prognosis of patients with BLCA and reveal relationship of immune features in BLCA with different risk scores. The study also provided a possible indicator for targeted therapy.

## Introduction

Bladder urothelial carcinoma (BLCA) is a common urinary tract cancer with annual worldwide estimates for nearly 500,000 new cases and 200,000 deaths. Ninety percent of bladder cancer is localized tumor, which is categorized into non–muscle-invasive bladder cancer (NMIBC, 75%) and muscle-invasive bladder cancer (MIBC, 25%) (Dy et al., 2017). Although NMIBC is literally not life-threatening, it still has a high rate of recurrence (50%–70%) even in patients accepting transurethral resection and combination therapies (Robertson et al., 2018). MIBC performs a poor prognosis with a 5-year survival of no more than 50% due to early occult metastatic dissemination (Hurst and Knowles, 2018). Ranging from chronically managed noninvasive tumors to advanced stage diseases that require multidisciplinary team treatment, bladder carcinoma represents long-term spectra of disease progression. The long-term disease process often accompanies with chronic inflammation infiltration. Clinicians considered inflammatory reaction as body’s defense mechanism against tumor although it aroused inappropriate systemic reactions to malignancy such as fevers, cachexia, and B symptoms (Diakos et al., 2014). However, some studies revealed that inflammation could play a negative effect in tumorigenesis and recurrence.

Because the observation of leukocytes within tumors first suggested the relation between inflammation and cancer two centuries ago (Elinav et al., 2013), the concept of inflammation-induced tumorigenesis was now generally accepted, and cancer-related inflammatory response also played an important role in modulating tumor progression, immune escape, and remodeling of tumor inflammatory microenvironment (Greten and Grivennikov, 2019). Under the mediation of inflammation, inactivation of tumor suppressor genes leading to dysfunction of DNA repair or apoptosis was also a possible mechanism (Van Opdenbosch and Lamkanfi, 2019). Several inflammatory response–related pathways were also reported to contribute to the occurrence and metastasis of bladder cancer (Shalapour and Karin, 2019). Numerous studies concentrated to look for a BLCA prognostic marker with high sensitivity and specificity. Too much attention was paid to establish scales on account of acute phase proteins and inflammation factors to better evaluate tumor prognosis. However, the correlation between IRGs and outcome of BLCA remains unclear. To fill the gap in this field, we constructed a prognostic signature and validated its stability and reliability in predicting the prognosis and overall survival (OS) in BLCA cohorts. We further performed functional enrichment analysis and immunity-related verification to explore the potential value of prognostic model in different pathological states. We also refined and supplemented our study by synthetically analyzing signature gene set together with tumor microenvironment (TME) formation and chemoresistance.

## Methods and Materials

**FIGURE 1:**
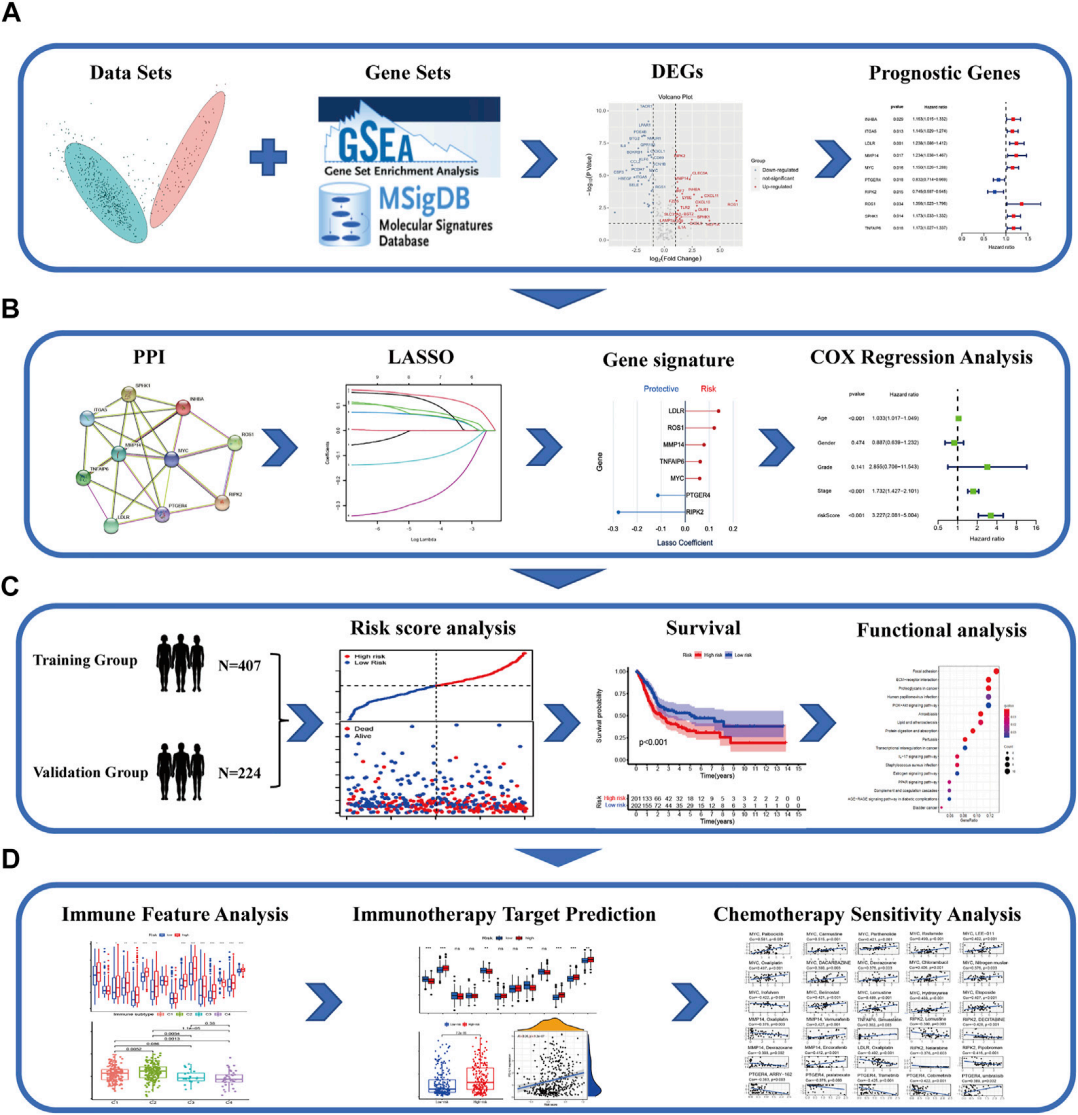
Schematic chart of the research flow. **(A)** The inflammatory response–related DEGs between the tumor and normal tissues in TCGA–BLCA dataset. Prognostic signature was established by univariate cox regression analysis on the basis of the DEGs. **(B)** PPI network was constructed to illustrate the interaction of gene-related proteins. LASSO-penalty cox analysis was executed for an optimized prognostic signature. **(C)** Comprehensive methods to validate the forecast performance of the IRG signature in the multiple databases. Functional enrichment analysis and survival curves. **(D)** The relationship between the IRGs and immune feature, immunotherapy target prediction, and chemotherapeutic drug sensitivity in patients with BLCA.

The flow diagram of the study is developed in Figure 1.

### Data Acquisition

We retrieved the transcriptome and clinical profiles of training datasets from the The Cancer Genome Atlas (TCGA) database on the GDC data portal. The HTSeq-FPKM workflow was applied to compare all available bladder transitional epithelial carcinoma samples with normal bladder epithelium tissues. Then, we downloaded the validation datasets containing RNA sequencing data with expression profiles and clinical information from the Gene Expression Omnibus (GEO) database to verify the constructed model. We totally gathered 414 tumor samples and 19 normal tissues from the TCGA and 308 tumor samples from GEO (GSE32894). Then, 200 IRGs that drive, suppress, or mark inflammatory response were acquired from the gene set HALLMARK_INFLAMMATORY_RESPONSE (M5932) in the MsigDB molecular signature database, which are shown in Supplementary Table S1. The Human Protein Atlas database was used to observe immunohistochemistry (IHC) staining of genes with prognostic values.

### Analysis of Differentially Expressed Genes

Differentially expressed genes (DEGs) between BLCA and normal bladder tissues were classified and screened out by R package “limma” with a |log2 fold change (FC)| > 1 and a false discovery rate (FDR) < 0.05 in TCGA cohort. The information of sample sources (TCGA–BLCA) was included as covariates in the analysis. All screened out genes were included in the univariate Cox analysis to clarify the relationship between multiple risk factors and gene-related prognosis.

### Establishment and Validation of a Prognostic IRGs Signature

The LASSO algorithm was used to minimize the overfitting risk with “glmnet” R package, so as to adjust some regression coefficients return to zero. In LASSO regression, the independent variable was normalized expression matrix of candidate prognostic genes. The dependent variable was the OS of patients in the TCGA cohort. The 10-fold cross-validation was used to determine the penalty parameter of the prognostic model and was followed the minimum criteria. The risk scores of patients were weighted calculated according to the expression quantity of each IRG and its corresponding regression coefficient. The formula was as follows:

$$Riskscore=\overset{n}{\underset{i=1}{\sum }}Coef\left(x_{i}\right)\times \;Exp\left(x_{i}\right)$$

In this formula, Coef (Xi) stood for the coefficient of each IRGs Xi, and Exp (Xi) stood for the expression levels of these genes. The risk scores were calculated and assigned to every individual. All involved individuals were assigned to the high- or low-risk groups, using the median risk score as a cutoff point. In line with the expressive abundance of genes in the signature, we used R package “Rtsne” and “ggplot2” to perform the PCA analysis and t-SNE analysis and thus to distinguish the distribution of individuals belong to different risk groups. The R package “survminer” was implemented to analyze the survival time of different cohort. The R package “survival” and “timeROC” were used to make time-independent receiver operating characteristic (ROC) curve to examine the efficacy of the prognostic model. Furthermore, Cox analyses were applied for selection of independent prognosis indicators.

### Functional Enrichment Analysis

Gene Ontology (GO, c5. go.v7.4. symbols) and Kyoto Encyclopedia of Genes and Genomes (KEGG, c2. cp.kegg.v7.4. symbols) analyses were performed with the “clusterProfiler” R package on the basis of signature genes using gene set enrichment analysis (GSEA) (version 4.1.0) software. Multiple GSEA analysis was completed to integrate the result of GO and KEGG analyses with “ggplot2” and “grid” R package.

### Immune Status Analysis

The “GSVA” R package was used to perform a single-sample GSEA (ssGSEA) to clarify the immune scores of included samples. The correlation between immune scores and risk classification was calculated by operating R package “limma”. To visualize the relationship between filtration of immune cells and relevant immune signal pathways, box plots were drew with R package “ggpubr” and “reshape2”. The association between risk scores and immune infiltration subtype was tested by two-way ANOVA analysis and performed with “ggpubr” R package. The infiltration of immune cells was correlated with risk scores in different methods, including TIMER, CIBERSORT, CIBERSORT-ABS, QUANTISEQ, MCPCOUNTER, XCELL, and EPIC.

### Analysis of Tumor Microenvironment and m6A Regulatory Factors

The infiltration of immune cells and stromal cells in tissues of different samples were calculated and named as TME scores, including immune score and stromal score. The correlation between risk scores and TME scores was explored with spearman correlation analysis. The potential connection between risk groups and expression of common m6a epigenetic regulatory factors was calculated by R package “ggplot2” and “ggpubr”.

### Chemotherapy Sensitivity Analysis

To examine whether the genes in signature were closely relevant to the drug sensitivity of chemotherapy, we utilized the NCI-60 database containing 60 different cancer cell lines from nine different types of tumors (Supplementary Table S2) and accessed through the CellMiner interface. Pearson correlation analysis was performed to investigate the correlation, which was visualized by C-map. Correlation analysis curves were built up with the efficacy of 218 drugs approved by Food and Drug Administration or in clinical trials (Supplementary Table S3).

### Statistical Analysis

R software (version 4.0.2) was applied to perform all statistical analyses and output visualization. Wilcoxon test was used to compare the DEGs between the pathological and normal bladder tissues. Univariate and multivariate Cox proportional hazard regression analyses were conducted to assess the association between risk score and prognosis. The ssGSEA immunity scores between the high- and low-risk groups were compared by Mann–Whitney test, while the *p*-values adjusted by the Benjamini–Hochberg method.

## Results

### Identification of Inflammatory Response–Related DEGs

**FIGURE 2:**
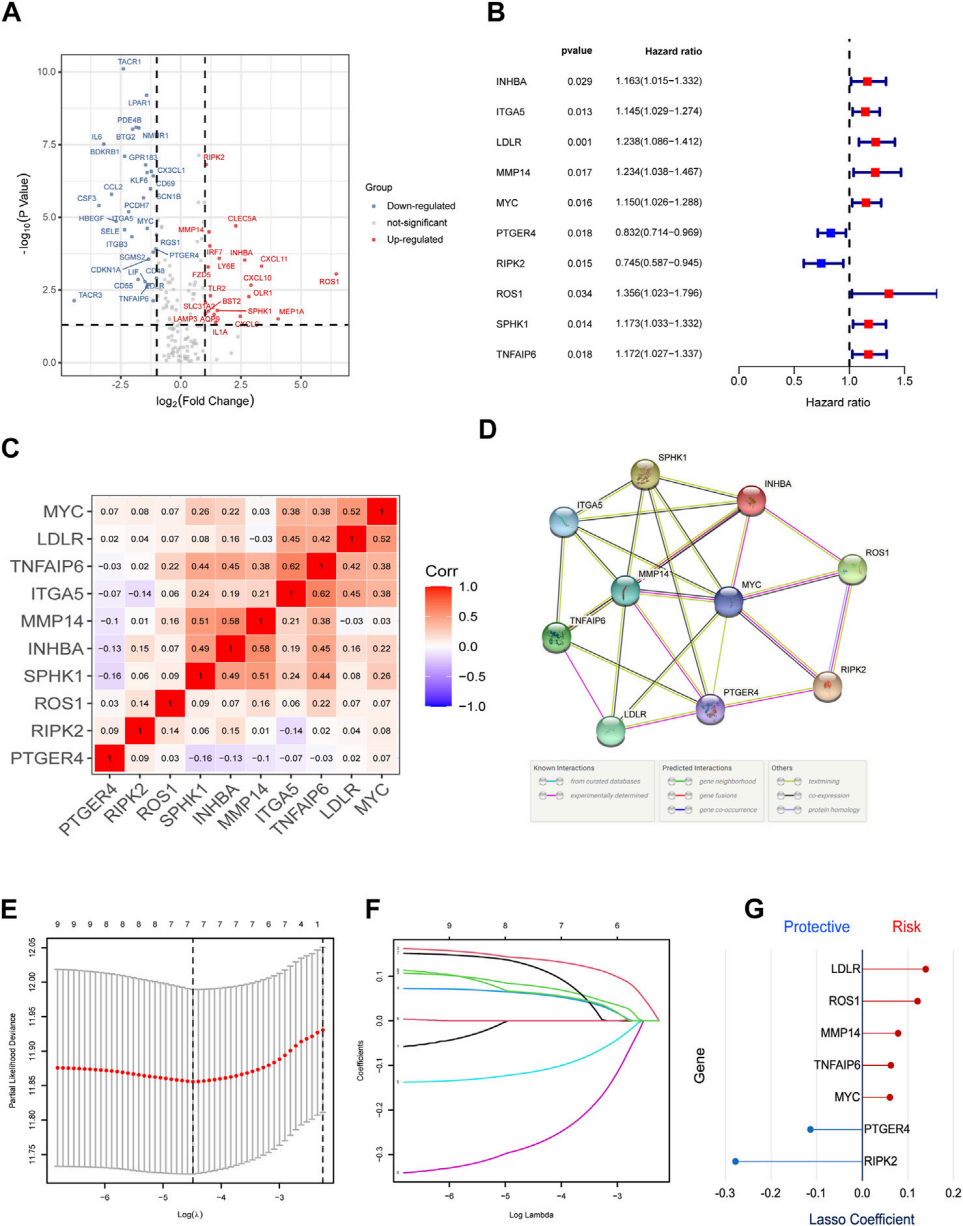
Identification of the candidate IRGs within the TCGA cohort. **(A)** Volcano plot to identify DEGs between bladder cancer tissues and normal tissues. **(B)** Univariate Cox Regression to screen out prognostic signature gene sets. **(C,D)** C-MAP and PPI network. **(E-G)** LASSO regression analysis to optimize the signature.

The study gathered a cohort of BLCA containing 414 tumor tissues and 19 normal tissues from TCGA database. Then, the validation group was constructed on the basis of GSE32894, which consists of 308 tumor tissues. Samples without survival time were eliminated and the amounts of the cohort were 407 in TCGA dataset and 224 in GSE32894. Some of the important clinical features of these patients are summarized in Table 1. The TCGA–BLCA cohort was set to be the trained group, whereas the validation group consolidated the GSE32894. Because we have extracted the expression values of 200 IRGs in patients with BLCA, 18 upregulated genes and 32 downregulated genes were authenticated (FDR < 0.05, log2 FC > 1). The differentially expressed IRGs were visualized *via* volcano plot (Figure 2A).

**TABLE 1:** Clinical Characteristics of selected patients with BLCA.

	TCGA–BLCA	GSE32894
No. of patients	407	224
Age (median, range)	69 (34–89)	70 (20–93)
Gender (%)		
Female	106 (26.04%)	61 (27.23%)
Male	301 (73.96%)	163 (72.77%)
Status (%)		
Alive	229 (56.27%)	199 (88.84%)
Dead	178 (43.73%)	25 (11.16%)
OS days (median)	536 (13–5,050)	1,052.5 (6–3,309)
Grade (%)		
High Grade (G2–G3)	384 (94.35%)	177 (79.02%)
Low Grade (G1)	20 (4.91%)	45 (20.09%)
Unknown	3 (0.74%)	2 (0.89%)
Stage (%)		
Stage I	2 (0.49%)	NA
Stage II	129 (31.70%)	NA
Stage III	141 (34.64%)	NA
Stage IV	133 (32.68%)	NA
Unknown	2 (0.49%)	NA

### Construction of Inflammatory Response–Related Prognostic Signature

First, we excluded seven samples without complete clinical information, which was necessary for the subsequent analysis in the TCGA training group. Then, we used univariate Cox regression to screen out 10 IRGs as prognosis-related gene (Figure 2B). The construction of the gene–gene correlation map (Figure 2C) and protein–protein interaction (PPI) network of these gene-coding proteins (Figure 2D) revealed the combination and interaction among these genes. To prevent the risk of overfitting and consequent deviation, we utilized the LASSO–Cox regression analysis to optimize the prognostic signature associated with the expression of the 10 genes above, in an attempt to avoid overfitting the model and the consequent deviation. Then, a better consolidated model consisted of seven core genes was established by the minimum value of lambda (λ) (Figures 2E–G). The gene set was divided into protective genes (PTGER4 and RIPK2) and risky genes (LDLR, ROS1, MMP14, TNFAIP6, and MYC) on the basis of hazard ratio (HR). The details of these genes are summarized in Supplementary Table S4. Supplementary Figure S1 shows the relationship between the survival and screened IRGs. We further established a risk scoring model to facilitate the assessment and prediction of prognosis and long-term progression. The risk classification was demarcated by the median value of the whole population.

### Evaluation and Validation of IRGs Signature

**FIGURE 3:**
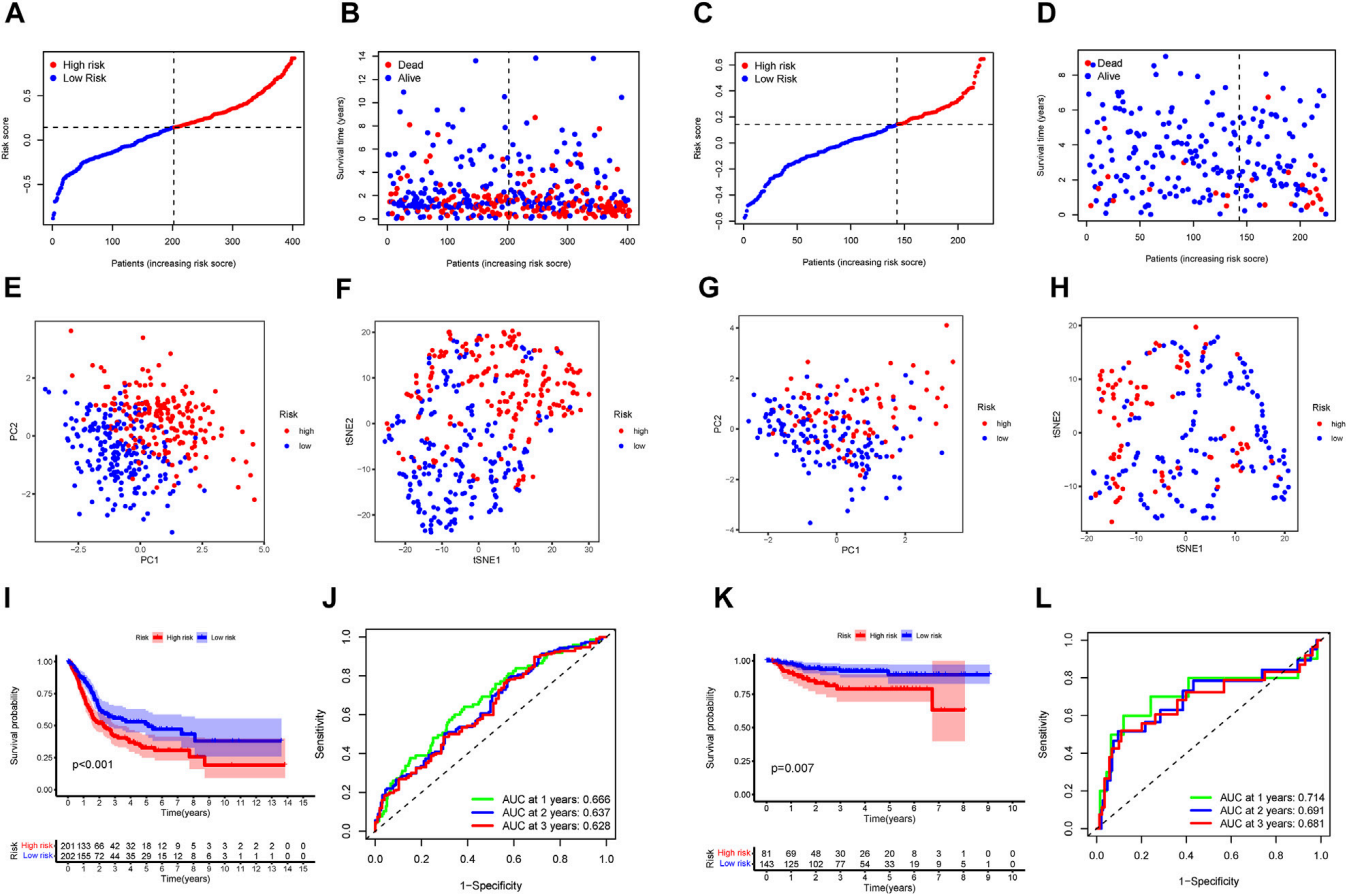
The IRGs prognostic model constructed with the TCGA cohort and GSE32894. TCGA cohort **(A, B, E, F, I, J)**. GSE32894 cohort **(C, D, G, H, K, L)**. **(A, C)** The median value and distribution of the risk scores. **(B,D)** The distribution of OS status. **(E,G)** PCA plot. **(F,H)** t-SNE analysis. **(I,K)** Kaplan–Meier curves for OS of patients in the high- and low-risk groups. **(J,L)** AUC time-dependent ROC curves for OS.

**FIGURE 4:**
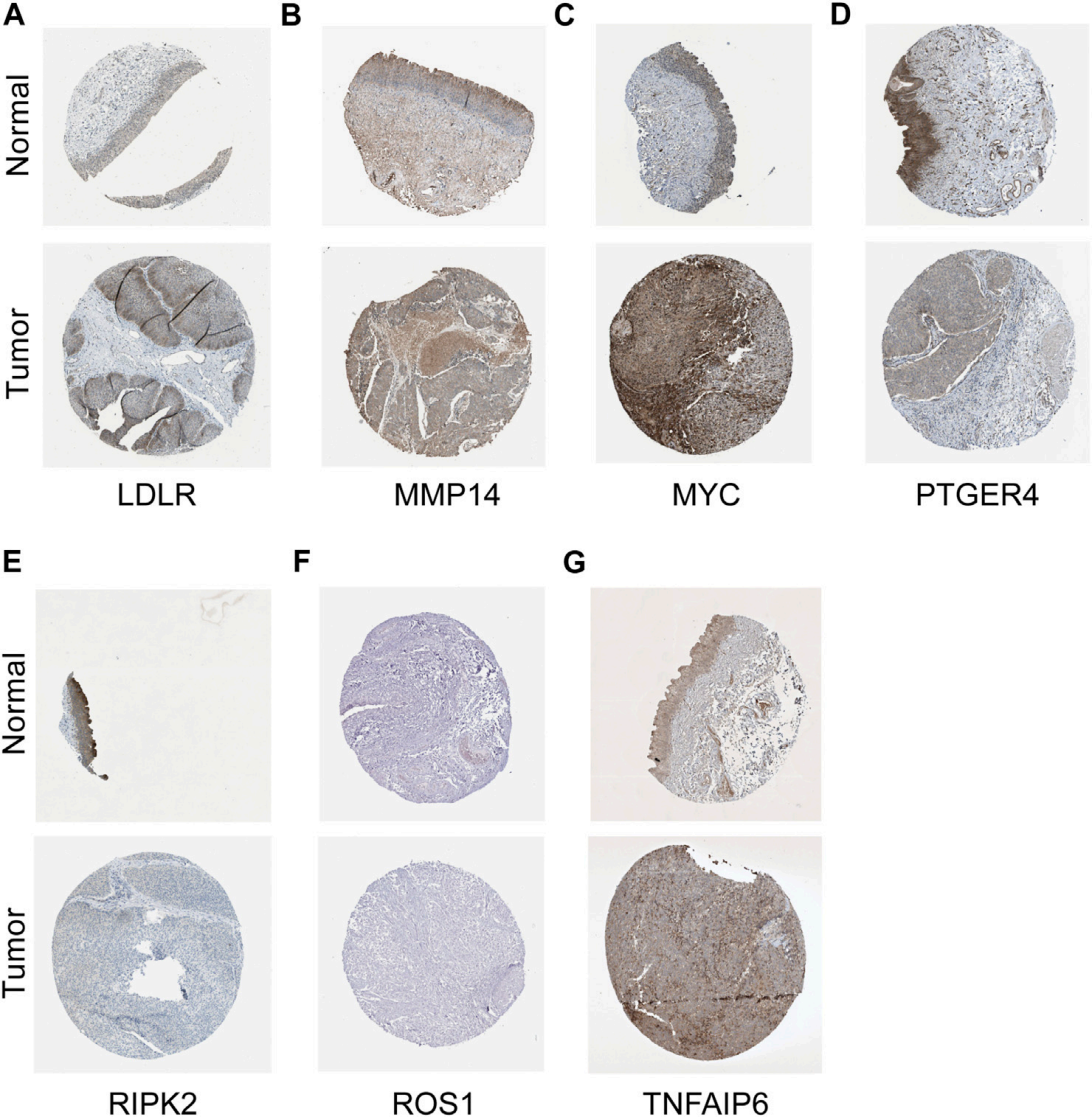
IHC staining in validating expression levels of the signature genes. LDLR **(A)**, MMP14 **(B)**, MYC **(C)**, PTGER4 **(D)**, RIPK2 **(E)**, ROS1 **(F)**, and TNFAIP6 **(G)** in normal tissue and tumor tissues.

To verify the reliability and feasibility of the established prognostic signature, we drew a scatter plot to illustrate the distribution of risk scores (Figure 3A) and the correlation between risk scores and OS (Figure 3B) in the training group. The individuals in the low-risk group appeared to have apparently lower probability to encounter death ending than that in the high-risk group. We downloaded the GSE32894, which contained 308 tumor tissues, to reconfirm the conclusion *via* the same demarcation (84 samples were abandoned for lack of survival and status data) (Figures 3C,D). The clinical stratification analysis was used to explore whether the IRG signature could be widely applied to predict the survival and prognostic conditions in the age (≤60 and >60), gender (male and female), and TMN stage. The results indicated that the individuals in the low-risk group might perform a better prognosis than that in the high-risk group for every subgroup (*p* < 0.01, Supplementary Figures S2C-I). PCA and t-SNE plot illustrated that patients were distributed into two sections (Figures 3E–H). The survival curves showed a better survival in the low-risk cohort (*p* < 0.001) in the training group, whereas the validation group also support this conclusion (*p* < 0.01, Figures 3I,K). The ROC curves were used to evaluate the efficiency of risk scores in predicting the OS, and the area under the curve (AUC) of the seven-gene model was 0.666 in 1 year, 0.637 in 2 years, and 0.628 in 3 years. In the validation group, this value was 0.714 in 1 year, 0.691 in 2 years, and 0.681 in 3 years (Figures 3J, L). Consistently, the IHC staining also verified the expression level of signature genes in normal and tumor tissues. (Figure 4).

### Independent Prognostic Value of the Signature in Training and Validation Cohorts

**FIGURE 5:**
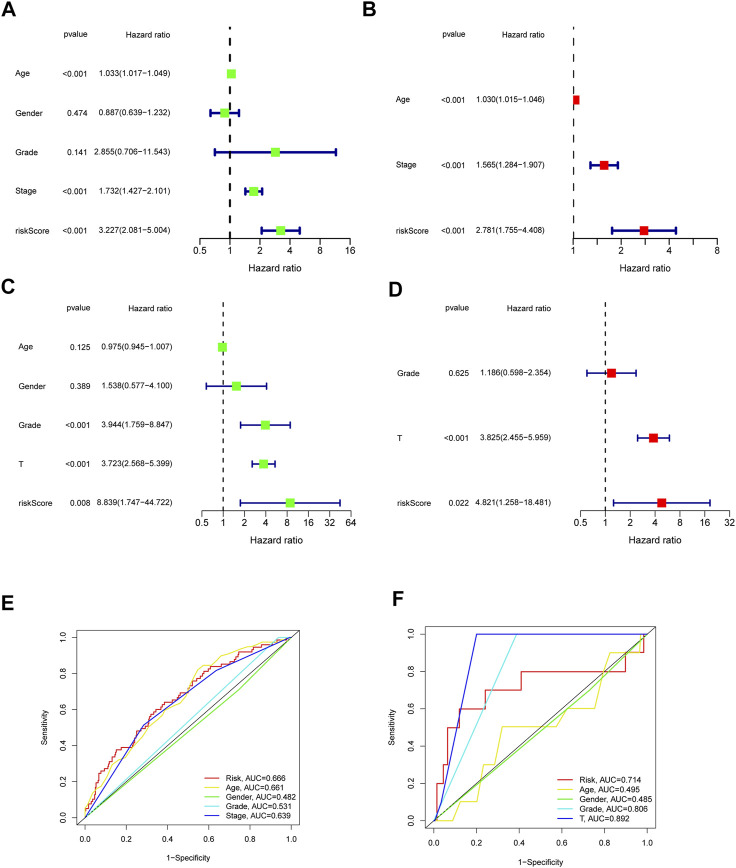
Outcome of Cox regression analysis with regard to survival time in the TCGA cohort **(A,B)**, GSE32894 **(C,D)**. Time-dependent ROC curve **(E,F)** to compare the prognostic accuracy of risk score, age, gender, tumor stage, tumor grade, and T Stage.

Furthermore, we analyzed the correlation between the risk scores and clinical characteristics of patients with BLCA by univariate and multivariate Cox regression analysis. The ability of the signature in predicting prognosis was independent. The univariate Cox regression identified the risk score [*p* < 0.001, HR = 3.227, 95% confidence interval (CI) = 2.081–5.004] and related clinical parameters including stage (*p* < 0.001, HR = 1.732, 95% CI = 1.427–2.101), grade (*p* = 0.141, HR = 2.855, 95% CI = 0.706–11.543), gender (*p* = 0.474, HR = 0.887, 95% CI = 0.639–1.232), and age (*p* < 0.001, HR = 1.033, 95% CI = 1.017–1.049) were, respectively, associated with OS in the TCGA training cohort (Figure 5A). Multivariate Cox regression analysis revealed that the risk score (*p* < 0.001, HR = 2.781, 95% CI = 1.755–4.408) was the independent prognostic factor of the OS (Figure 5B). Besides, similar to the results of the training group, the risk score (*p* = 0.022, HR = 4.821, 95% CI = 1.258–18.481) in the GSE32894 validation cohort was also proven to be the independent prognostic factors influencing the OS (Figures 5C,D; Table 2). The ROC curves were drawn to evaluate efficiency of the Cox regression analysis (Figures 5E,F). According to univariate and multivariate Cox regressions, we built a nomogram (Iasonos et al., 2008) to assess the probability of 1-, 2-, and 3-year OS of patients (Supplementary Figures S2A, 2B).

**TABLE 2:** Univariate and multivariate Cox regression analysis in TCGA and GSE32894 cohort.

Variables	Univariate analysis			Multivariate analysis		
	HR	95% CI	*p*-value	HR	95% CI	*p*-value
TCGA–BLCA						
Age	1.033	1.017–1.049	<0.001	1.030	1.015–1.046	<0.001
Gender	0.887	0.639–1.232	0.474			
Grade	2.855	0.706–11.543	0.141			
Stage	1.732	1.427–2.101	<0.001	1.565	1.284–1.907	<0.001
Risk score	3.227	2.081–5.004	<0.001	2.781	1.755–4.408	<0.001
*GSE32894*						
Age	0.975	0.945–1.007	0.125			
Gender	1.538	0.577–4.100	0.389			
Grade	3.944	1.759–8.847	<0.001	1.186	0.598–2.354	0.625
T Stage	3.723	2.568–5.399	<0.001	3.825	2.455–5.959	<0.001
Risk score	8.839	1.747–44.722	0.008	4.821	1.258–18.481	0.022

### Functional Enrichment Analysis of IRGs Signature

**FIGURE 6:**
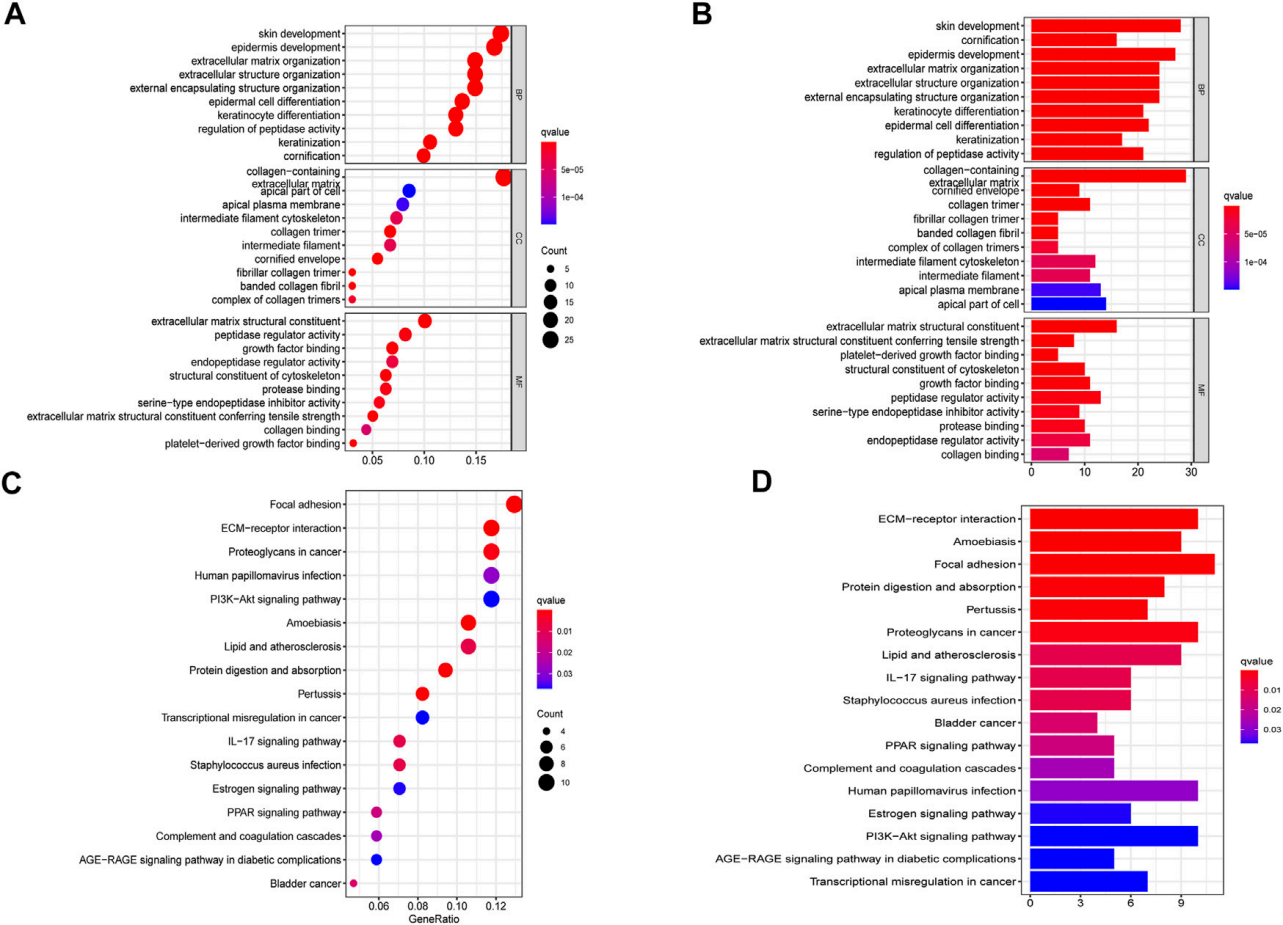
Gene set enrichment analysis of pathways and biological functions based on TCGA cohort. **(A,B)** GO, Gene Ontology. **(C,D)** KEGG, Kyoto Encyclopedia of Genes and Genomes.

To further explore the biological functions and mechanistic pathways that were correlated with IRGs in BLCA, the GO and KEGG analysis were performed between the high-risk and low-risk groups in both TCGA and GEO cohorts. Interestingly, several inflammatory response–related molecular functions were enriched, such as extracellular matrix (ECM) organization, human immune response, and antimicrobial humoral response (Figures 6A,B). In addition, IRG set mainly participated in several metastasis-related biological processes including focal adhesion, ECM–receptor interaction, and PI3K-Akt signaling pathway (Figures 6C,D). The integration of the GO enrichment and KEGG pathway analysis came to a conclusion that the seven-gene signature was closely related to metastasis and invasion of tumor. The same analysis was performed on the basis of the GSE32894 dataset to verify the accuracy of the trained group. The result indicated that the IRG set was linked to *epidermis* development, cornification, and IL-17 signaling pathway (Supplementary Figure S3). Multiple GSEA analysis was utilized to explore the potential biological mechanisms of the inflammatory response–related signature involved in BLCA progression (Supplementary Figure S4).

### Immune Feature and Tumor Microenvironment Analysis of IRGs Signature

**FIGURE 7:**
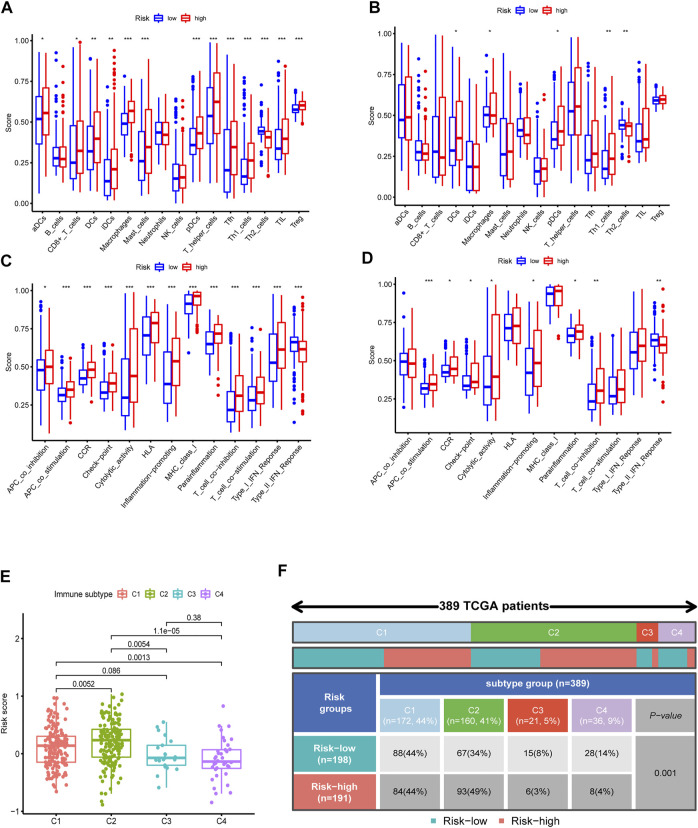
Immune feature analysis and immune subtype classification. Comparison of immune cells **(A**, TCGA; **B**, GSE32894**)** and immunologic function **(C**, TCGA; **D**, GSE32894**)** between the high- and low-risk groups. **(E,F)** Immunologic subtypes correlated with risk scores.

**FIGURE 8:**
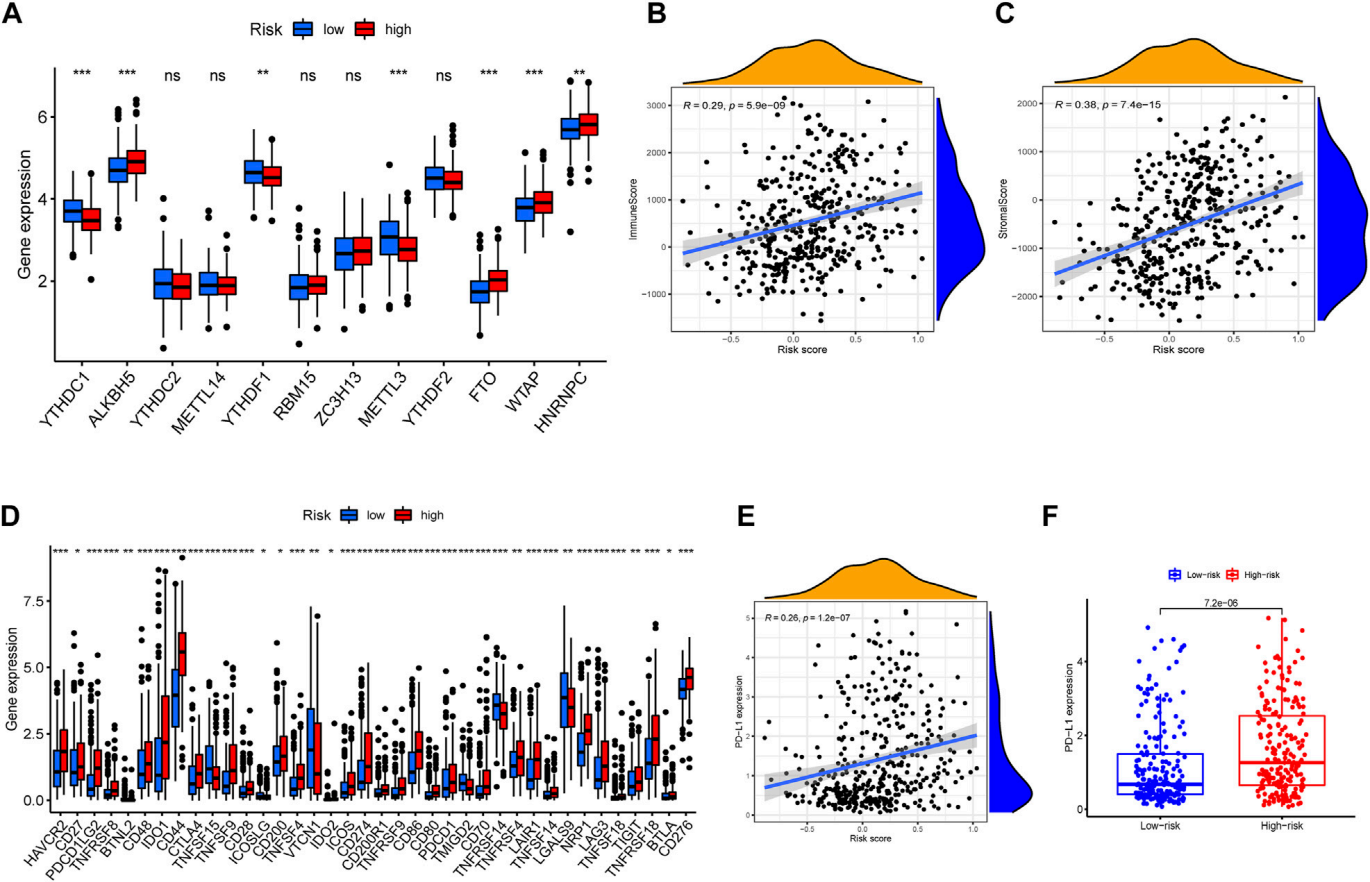
m6a correlation analysis and immune checkpoint correlation analysis. **(A)** Expressions of m6a-related genes in different risky groups. **(B,C)** The correlation between tumor microenvironment feature and risk scores. **(D)** Gene expression of immune checkpoint sites in distinct risk groups. **(E)** Correlation analysis between risk scores and expression level of PD-L1 gene. **(F)** Comparison of PD-L1 expression levels between high- and low-risk groups.

To further investigate the immune feature in the high- and low-risk subgroups, the ssGSEA was performed to analyze the differences in the immune cell infiltrations and immune signal pathways. It was found that, in the high-risk group, the immune scores in DCs, macrophages, pDCs, Th1 cells, APC co-stimulation, and T cell co-inhibition were significantly higher. The score of Th2 cells and type Ⅱ interferon (IFN) response was significantly decreased in the high-risk group (Figures 7A–D). Subsequently, we explored the correlation of risk scores with immune infiltration, to confirm how risk score was associated with immune subtypes. Previous studies revealed that there are six primary types of immune infiltrations that were identified in human solid tumors. These subtypes, ranging from tumor progression to tumor suppression, respectively, were named as C1 (wound healing), C2 (INF-g dominant), C3 (inflammatory), C4 (lymphocyte depleted), C5 (immunologically quiet), and C6 (TGF-b dominant) (Tamborero et al., 2018). In the correlation plot of our study, almost all samples were concentrated from C1 to C4. Because no sample from TCGA–BLCA was matched to C5 and C6 immune subtypes, these two immune subtypes were excluded from the study. In addition, most individuals in the high-risk group aggregated in the subtype C1 and C2 (totally 93%) (Figures 7E,F), which indicated that C1 and C2 subtypes were correlated with poor prognosis. This result partially provided a brand-new research tendency and therapeutic guideline for immune infiltrated tumors. The infiltration of common immune cells was showed in heatmap on the basis of TCGA dataset (Supplementary Figure S5). As stromal cells play an essential role in the construction of the TME, particularly in MIBC, we conducted a correlation investigation of prognostic genes in the immune microenvironment. The IRG signature was found to be closely related to the stomal score of BLCA, indicating that the IRG sets were significantly positively characterized in relation to the TME formation (Figures 8B,C).

### Expression of Immune Checkpoint Gene and m6a-Related Target Site

Then, we investigated the relevance of IRG signature to the content of immune checkpoint gene expression levels. In particular, PD-L1, as a key regulator in cancer immune evasion and surveillance, was an important indicator to guide individualized immunotherapy. As shown in Figures 8E,F, the risk score was significantly positively correlated with PD-L1 expression (r = 0.26, *p* < 0.001). The expression level of similar immune checkpoint genes was partly correlated with effect of immunotherapy. The result of immune checkpoint analysis revealed as expected that the expression of some famous targets was significantly higher in the high-risk group compared with the lower one (Figure 8D). Furthermore, we also investigated the relationship between m6a-related genes and risk scores. The m6A modifications were involved in almost all aspects of RNA metabolism, and transcriptome analysis of m6A-containing genes also revealed that the most relevant functional pathways were related to RNA metabolism (Sun et al., 2019). Previous studies revealed that m6a modification also greatly affected tumor progression. The correlation plots showed that the expression of m6a-related genes was elevated in the high-risk group (Figure 8A), illustrating that the signature was partly connected with epigenetic modification and took role in tumor progression and evolution.

### The Relationship Between IRG Signature and Clinical Drug Resistance

**FIGURE 9:**
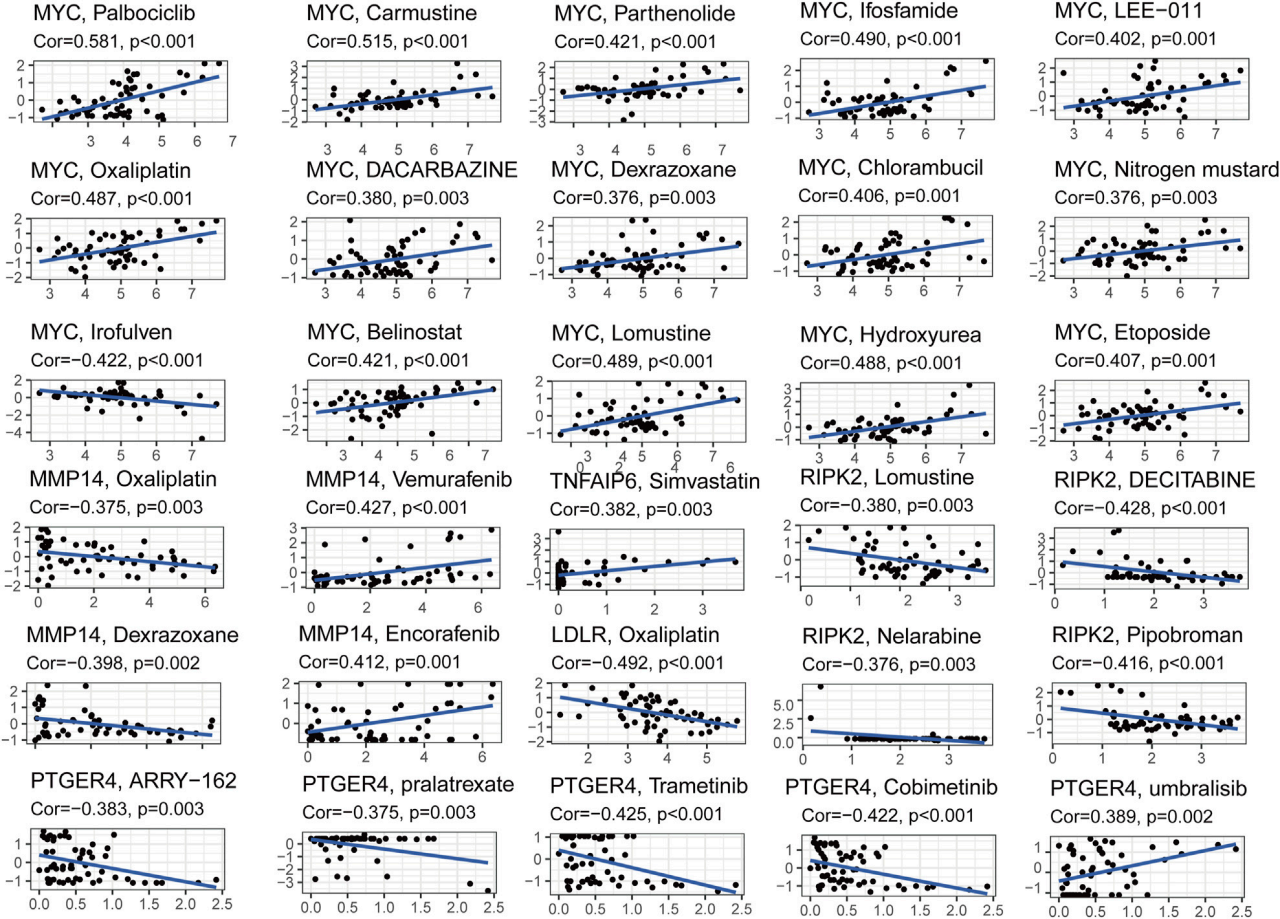
Scatter plots of correlation analysis between chemotherapeutic drugs sensitivity and expression of signature genes.

To explore the relationship between the seven-gene signature and the BLCA drug resistance, we built the Scatter plots reflecting the expression distribution of the seven genes in some drug resistance datasets. Then, we investigated the expression of signature genes in NCI-60 cell line. The results revealed that high expression of MYC, MMP14, TNFAIP6, and PTGER4 were correlated with drug sensitivity of cancer cells to a multitude of chemotherapeutic drugs, especially *MYC*, which was highly correlated with Palbociclib, Carmustine, Ifosfamide, Parthenolide, etc. Conversely, increased expression of *MMP14*, *RIPK2*, *LDLR*, and *PTGER4* was associated with increased drug resistance of cancer cells to Oxaliplatin, Lomustine, Decitabine, Dexrazoxane, trametinib, etc. (Figure 9).

## Discussion

In this study, we established a prognostic model with a signature of IRGs, and the model could efficiently identify the degrees of survival risk and predict the bladder cancer prognosis. Consistent with previous studies of bladder cancer, we found that the outcome was closely connected with inflammatory response. However, rare study focused on the prognostic value of IRGs in bladder cancer. Furthermore, lack of biomarker or other signature may contribute to the misuse and overuse of pharmacological therapy, which can arouse the risk of tumor recurrence and drug resistance. Therefore, it is necessary to identify the crucial IRGs that reflected the relationship between inflammation and BLCA prognosis, thus helping directing clinical treatment.

We systematically analyzed the expression profile of 200 IRGs in public databases, combined clinical survival data with DEGs expression profile, and formatted a correlated matrix. Hence, 10 inflammatory response–related DEGs were selected, which were assumed to be significantly correlated with the prognosis of patients with BLCA. The signature was finally simplified to seven genes, of which functions were summarized in Supplementary Table S4. Notably, most of these genes in signature were documented to be close relevant to progression and metastasis. *PTGER4* was a receptor for PGE2 that mediated both inflammatory and regulatory eicosanoid signaling (Roulis et al., 2020). Kinase inhibitor targeting *RIPK2* was reported to ameliorate NOD-mediated pathologies, indicating that *RIPK2* might mediate inflammatory response signaling by the bacteria-sensing receptors *NOD1* and *NOD2* (Roulis et al., 2020)*. TSG-6* (*TNFAIP6 encoding protein*) was documented to be involved in accelerating wound healing and reducing tissue fibrosis, and it could regulate chemokines by inhibiting the interaction of chemokines and mucopolysaccharides (Dyer et al., 2016). *ROS1*, as a proto-oncogene, might phosphorylate and activate the transcription factor *STAT3* to control anchorage-independent cell growth (Charest et al., 2006). *MYC* activation profoundly accelerated lung tumor progression at every stage of adenoma evolution (Shi et al., 2014), triggering a precipitous drop in survival. *MMP14* regulated the activity of multiple extracellular and plasma membrane proteins, influencing cell–cell and cell–ECM communication (Noda et al., 2003). This regulation mediated processes such as ECM degradation, remodeling, cell invasion, and cancer metastasis. The outcome of GSEA also supported that the continuous activation of tumor-related signalings such as ECM receptor interaction and glycerophospholipid metabolism were associated with the development of bladder cancer, which might provide some potential therapeutic targets for targeted therapy of bladder cancer. Inflammatory response–related pathways were significantly enriched in the high-risk group, elucidating that inflammatory response was closely associated with bladder cancer progression.

The correlation between risk score and clinical characteristics was also exhibited in the Supplementary Figure S2. Cohort with high-risk score was illustrated to be significantly associated with tumor grades 3–4 or tumor stages III–IV, indicating that high-risk score was definitely related with poor prognosis. However, the IRG signature genes were still lack of relevant research studies to explore or verify the function of these genes in BLCA.

Inflammatory response, as a vital part of systematic host immune reaction, played an important role in the process of early recruitment of inflammatory factors and formation of TME (Bonavita et al., 2020). Immune factors recruited in inflammatory response were also reported to be closely related with tumor necrosis and tumor distant metastasis (Quail and Joyce, 2013). Existing research suggested that IRGs not only participated in the activation of inflammatory response but also contributed to construction of TME (de Oliveira et al., 2019). Inflammatory response also participated the process of tumor surveillance (Grivennikov et al., 2010), immune evasion (Batlle and Massagué, 2019), and tumor dormancy (Baram et al., 2020).

Previous studies indicated that the host immune response killed tumor cells and inhibited tumor growth in the early stage of cancer. However, after the tumor evolved and developed into advanced stage, the establishment of TME and the formation of the trained immunity and immune tolerance indicated that immunity played a role in tumor development. Because inflammation occurred in the early stages of the host immune response, the correlation analysis also verified the previous point in relationship between TME and tumor.

Immune cells were widely involved in the initiation and progression of tumor. Both macrophages and dendritic cells were shown to play a deleterious role in immune escape and trained immunity of tumors. In the comparison of immune cell function, the functional characteristics of stimulating APC and IFN-γ immune regulatory response in the high-risk group were significantly higher than those in the low-risk group. Tumor developed multiple mechanisms to evade immune surveillance, including inhibition of DC function (Böttcher et al., 2018) and downregulation of HLA-1 expression by interfering with antigen processing (Qifeng et al., 2011). In the early tumor formation stage, high level of IFN-γ would transiently stimulate activation of the immune system and participate the TSG responses (Ikeda et al., 2002). However, chronic IFN-γ stimulation induced methylation of tumor DNA or gene mutations, which led to the tumor progression and recurrence (Glasner et al., 2018). Dialectically speaking, although the infiltration of immune cells might increase the risk of poor outcome in patients with BLCA, it also provided some potential therapeutic intervening measures. On the basis of the concept of “cold tumor” and “hot tumor”, the presence of immune infiltration in tumor tissue and the characteristic expression of immune cell function all determined the efficacy of tumor immunotherapy to a certain extent (Bonaventura et al., 2019).

Besides resection, immunotherapy and chemotherapy have also become very important component of the comprehensive tumor therapy. Targeting immune checkpoints such as anti–PD-L1 antibodies have been proved valid in different types of solid tumors. The curative effect of immunotherapy depended on the expression of immune checkpoint proteins (Postow et al., 2018). Increased immune checkpoint gene suppressed the anti-tumor immune response of T cells by increasing the expression of PD-1 and CTLA4 reporters. Chemotherapy was designed to compromise cellular integrity during division; however, these agents could also induce remodeling of immunity that either impeded or augmented overall treatment efficacy (Pinedo and Giaccone, 1997). MIBC was reported to benefit from targeted therapy and chemotherapy (Choi et al., 2014; Leow et al., 2014).

Our study then examined the potential prognostic value of the inflammatory response–related signatures in the response of commonly used drugs and drug resistance of chemotherapy in different databases, to overcome previous drug resistance or improve the toxicity of large dose drugs to provide a potential guideline for rational clinical use. Although the inflammatory response–related prognostic model was consolidated with different public datasets, some more prospective and updated data are still necessary. The signature only includes IRGs, and many genes for other traits that are of prognostic value may be excluded from the present study. Moreover, our study only preliminarily revealed the relationship between IRGs and some immune status or chemotherapeutic drugs sensitivity. The underlying mechanisms can be further explored by molecular mechanism experiments.

Briefly, this study does suggest a potential direction in predicting the prognosis and survival risk in patients with BLCA. One important future direction will be the early diagnosis and precision medicine of tumor. Thus, the research results will hopefully serve as useful feedback for improvement of previous tumor individualized treatment.

## Conclusion

To sum up, our study defined a novel prognostic signature consisting of 7-IRGs for patients with BLCA. The signature was validated feasible to evaluate immune status, immune checkpoints, TME formation, and chemotherapy drug sensitivity, providing an insight in predicting prognosis of BLCA and assisting therapeutic decision.

## Data Availability

Publicly available datasets were analyzed in this study. This data can be found here: The Cancer Genome Atlas database (TCGA-BLCA) (https://portal.gdc.cancer.gov/) and Gene Expression Omnibus database (GSE32894) (https://www.ncbi.nlm.nih.gov/geo/query/acc.cgi?acc=GSE32894).
