# Supplementary material for: Identification and Development of Inflammatory Response–Related Genes Signature Associated With Prognosis Evaluation and Immune Status of Bladder Cancer
Source: Front Cell Dev Biol. 2022 Mar 3;10:837849. doi: 10.3389/fcell.2022.837849 (PMC8927776; doi:10.3389/fcell.2022.837849)
Supplement: Supplementary file 2 [file DataSheet1.DOCX]

**Identification and Development of Inflammatory Response-Related Genes Signature Associated With Prognosis Evaluation and Immune Status of Bladder Cancer**

**Supplementary Figures:**


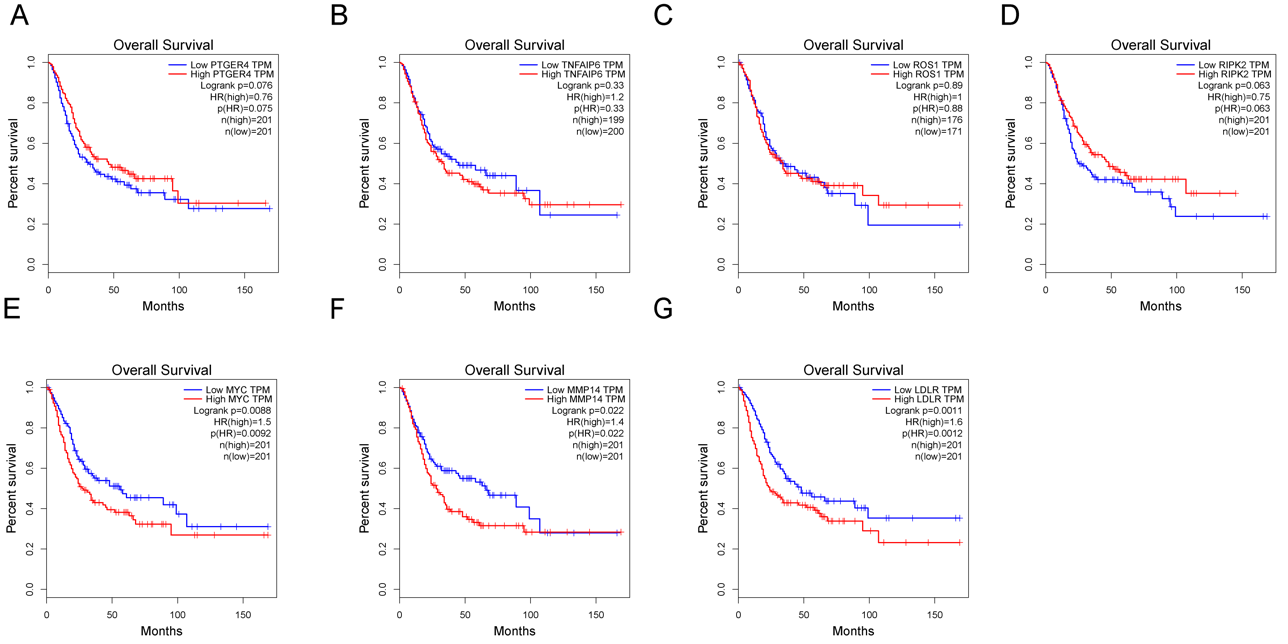


**Supplementary Figure 1.** Survival analysis of prognostic genes according to the median cut-off expression value in TCGA-BLCA cohort (A–G).


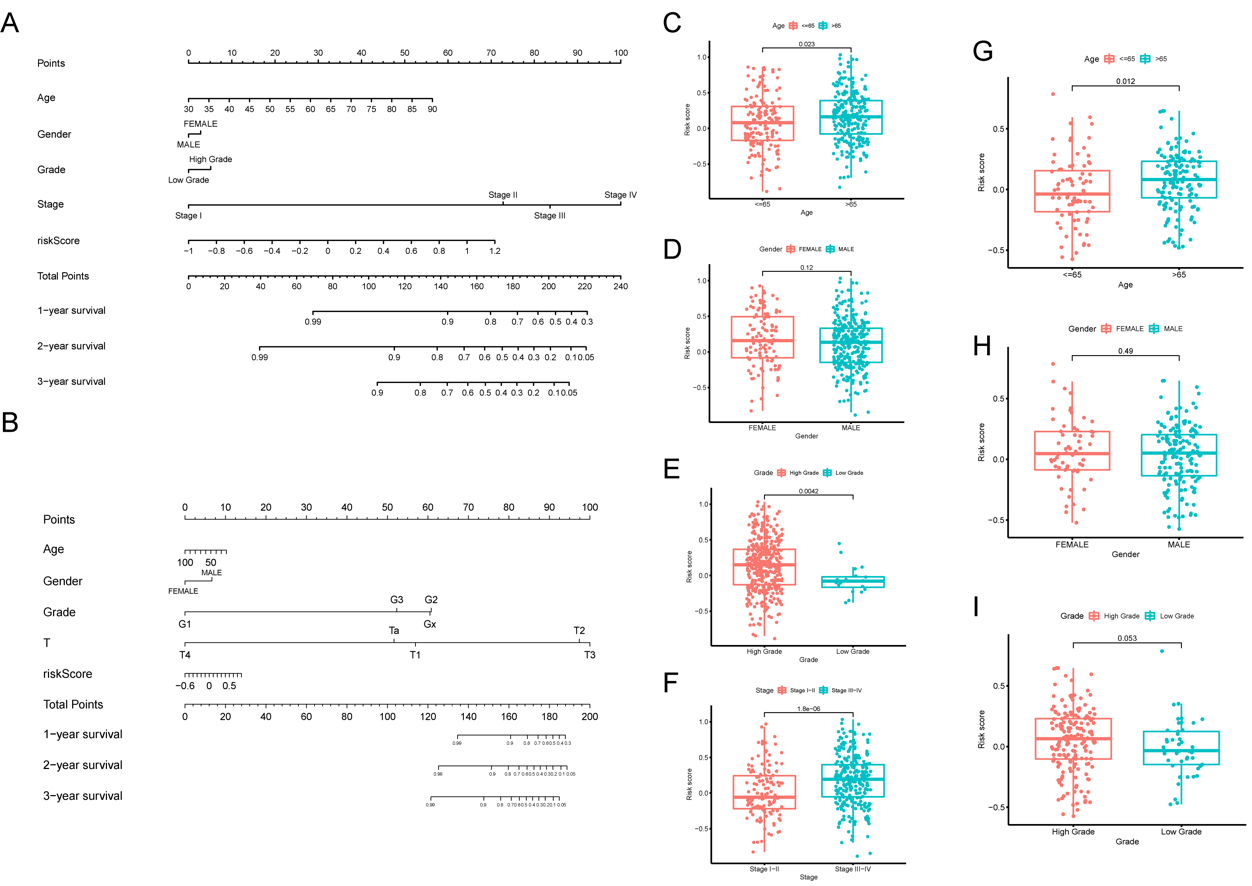


**Supplementary Figure 2.** Construction and validation of the nomogram to predict prognosis in (A) TCGA-BLCA and (B) GSE32894 dataset. The risk score in different groups divided by clinical characteristics. TCGA-BLCA: (C) Age, (D) Gender, (E) Grade, (F) Stage; GSE32894: (G) Age, (H) Gender, (I) Grade.


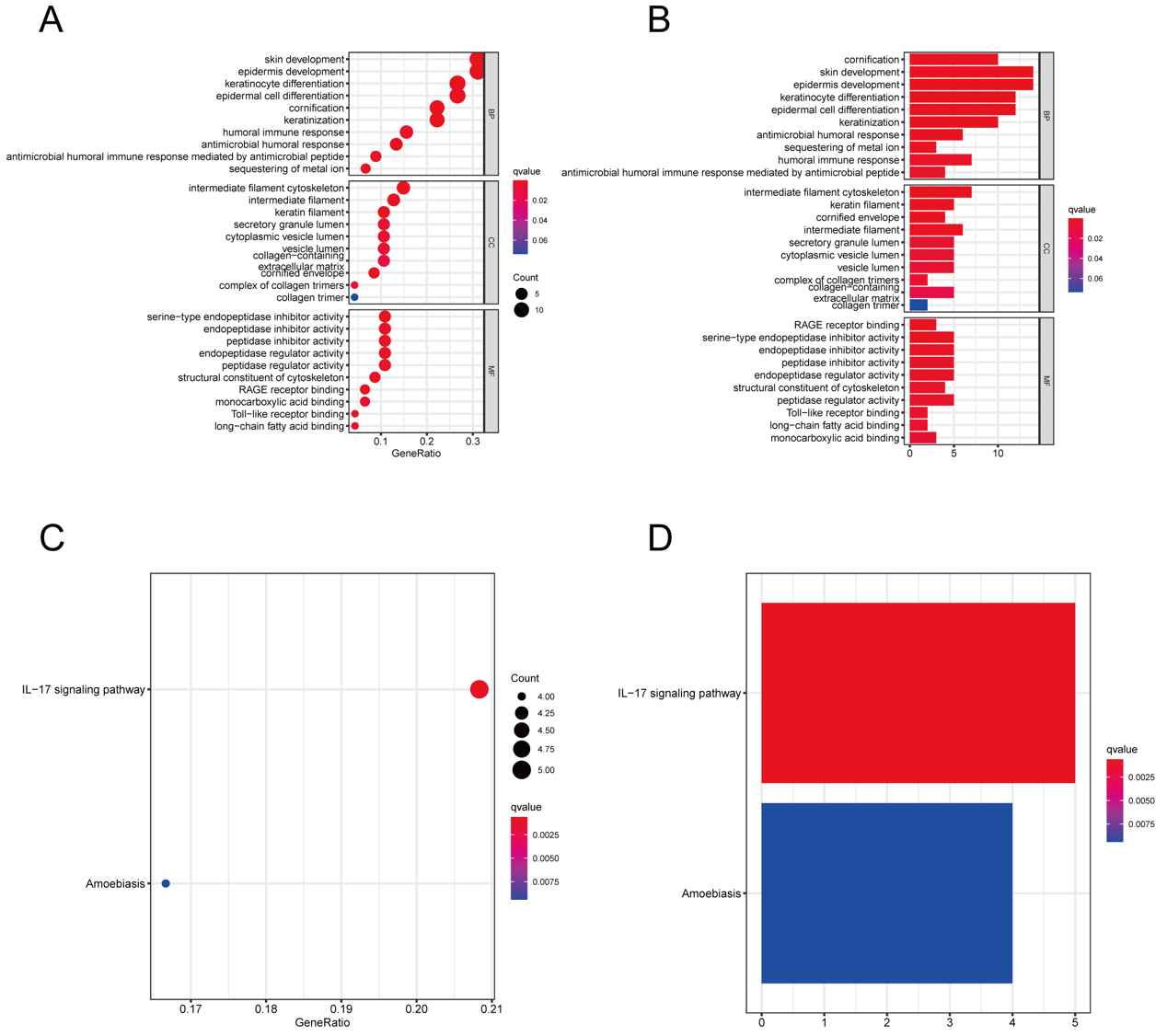


**Supplementary Figure 3.** Enrichment analysis of pathways and biological functions based on GSE32894 cohort. (A, B) GO, Gene Ontology. (C, D) KEGG, Kyoto Encyclopedia of Genes and Genomes.


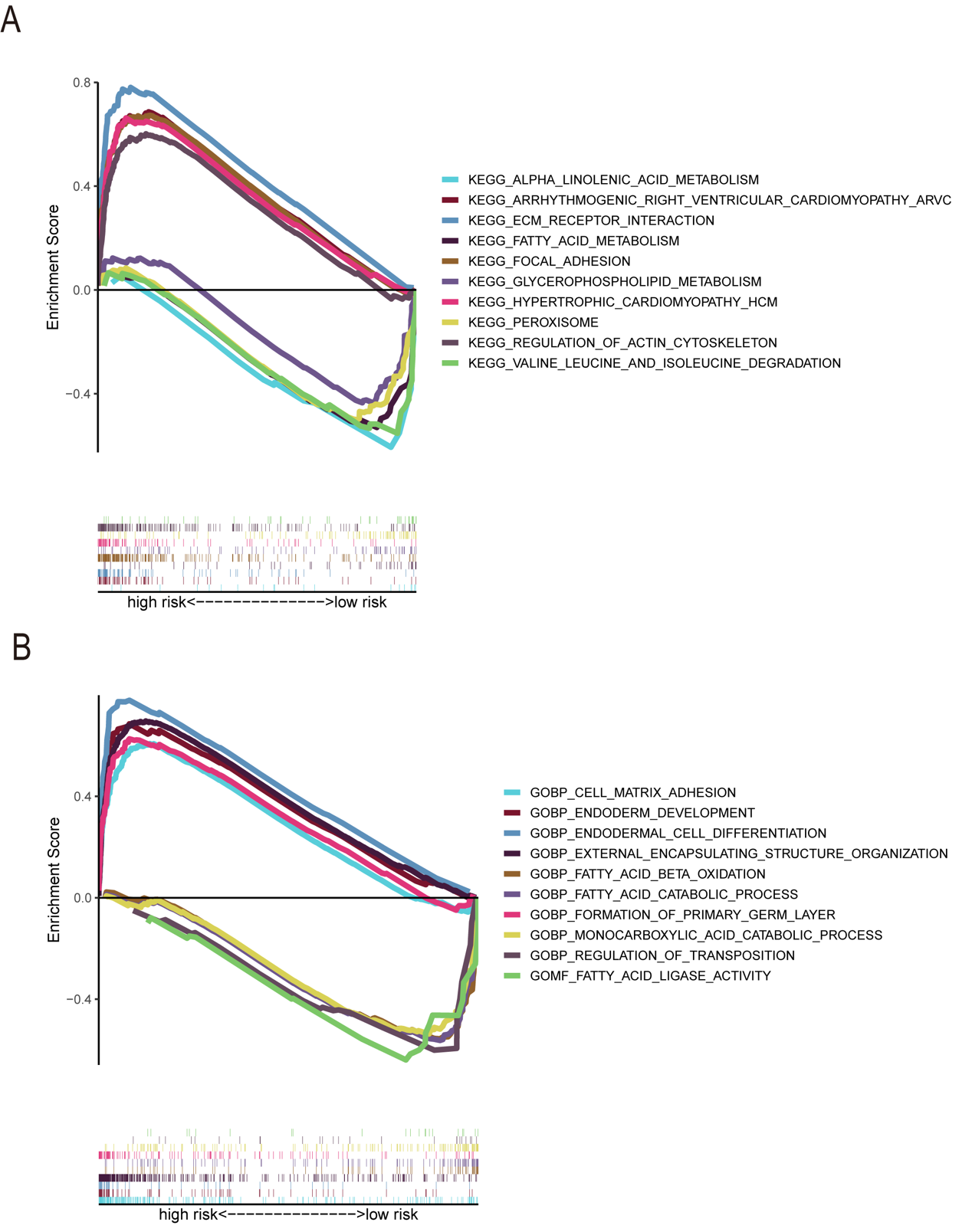


**Supplementary Figure 4.** Gene set enrichment analysis of Biological functions and pathways. (A) KEGG, Kyoto Encyclopedia of Genes and Genomes. (B) GO, Gene Ontology.


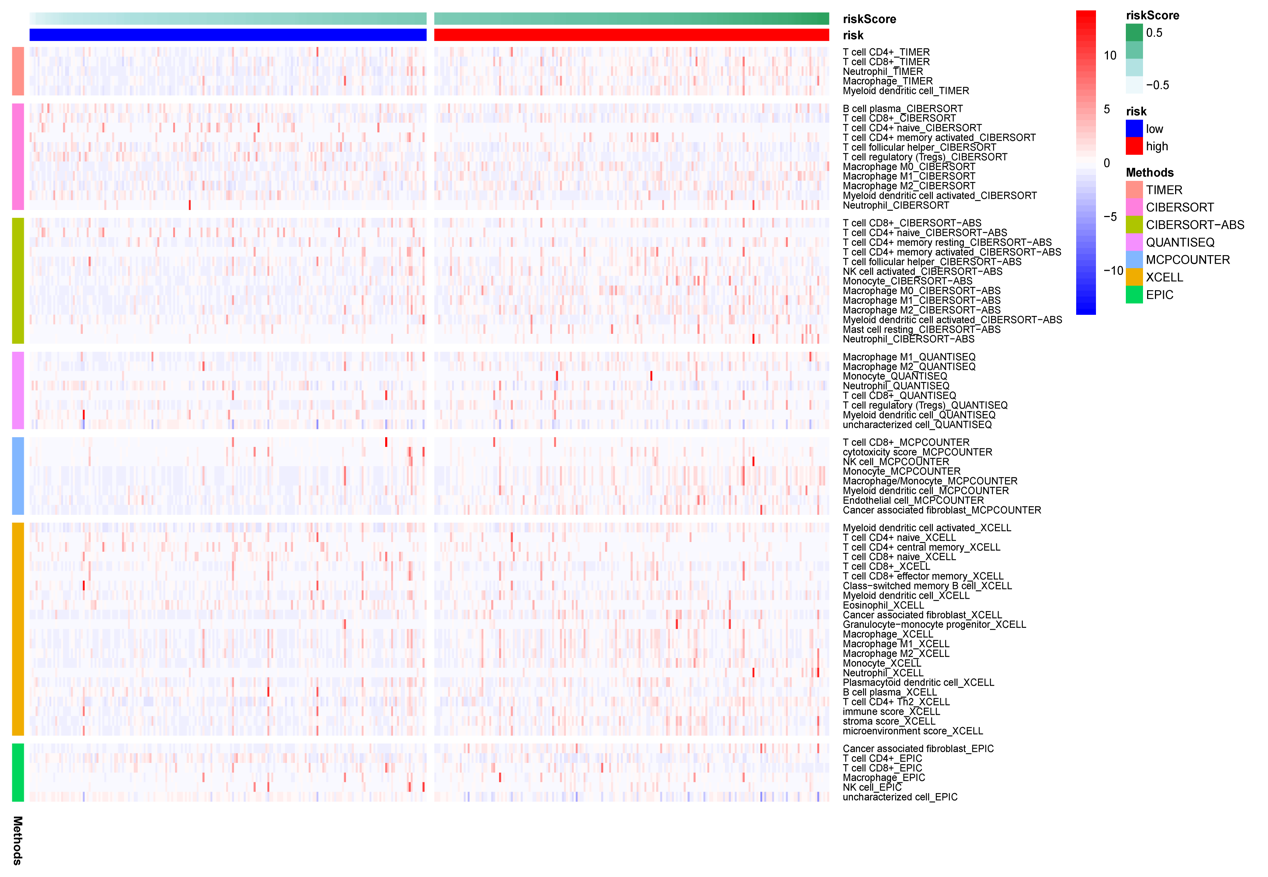


**Supplementary Figure 5.** Heatmap of the infiltration of common immune cells in the high- and low-risk groups.
